# QTL mapping reveals a tight linkage between QTLs for grain weight and panicle spikelet number in rice

**DOI:** 10.1186/1939-8433-6-33

**Published:** 2013-11-28

**Authors:** Xiao Luo, Shi-Dong Ji, Ping-Rong Yuan, Hyun-Sook Lee, Dong-Min Kim, Sangshetty Balkunde, Ju-Won Kang, Sang-Nag Ahn

**Affiliations:** Department of Agronomy, College of Agriculture & Life Sciences, Chungnam National University, Daejeon, 305-764 Korea; Nanyang Normal University, Nanyang City, 473061 Henan Province China; Institute of Food Crop Research, Yunnan Academy of Agricultural Sciences, Kunming, 650205 Yunnan China

**Keywords:** Rice, Spikelets per panicle, 1,000-grain weight, QTL, Linkage, Near isogenic lines

## Abstract

**Background:**

A number of QTL studies reported that one genomic region was associated with several traits, indicating linkage and/or pleiotropic effects. The question of pleiotropy versus tight linkage in these studies should be solved using a large-size population combined with high-density mapping. For example, if each of the 2 parents has a TGW-increasing or SPP-increasing QTL that is tightly linked, complementary combination of the 2 beneficial QTLs by using molecular markers could produce higher yields compared to the 2 parents. However, a pleiotropic QTL with opposite effects on the SPP and 1,000-grain weight (TGW) is complicated and challenging in terms of its application to rice improvement.

**Results:**

In this study, using a series of BC_5_F_4_ nearly isogenic lines (NILs) that were derived from a cross between the Korean *japonica* cultivar Hwayeongbyeo and *Oryza rufipogon*, we demonstrated that 2 QTLs, *qSPP5* for spikelets per panicle (SPP) and *qTGW5* for grain weight (TGW), are tightly linked on chromosome 5. Alleles from the *O. rufipogon* parent increased the SPP and decreased TGW in the Hwayeongbyeo background. *qSPP5* was located within a 803-kb interval between the simple sequence repeat (SSR) markers INDEL3 and RM18076. Based on the map position, *qTGW* 5 seemed to be the same gene as *qSW5*, which controls grain morphology. The additive effect of the *O. rufipogon* allele at *qSPP5* was 10–15 SPP, and 33.0% of the phenotypic variance could be explained by the segregation of the SSR marker RM18058. Yield trials with BC_5_F_4_ NILs showed that lines that contained a homozygous *O. rufipogon* introgression at the *qSPP5* region out-yielded sibling NILs that contained Hwayeongbyeo DNA by 15.3% and out-yielded the Hwayeongbyeo parent by 7.3%.

**Conclusion:**

Based on the finding that the *O. rufipogon* allele for the SPP was beneficial in the *japonica* and *indica* cultivar backgrounds, the *qSPP5* allele could be valuable for improving rice yields. In addition, the NIL populations and molecular markers are useful for cloning *qSPP5*.

**Electronic supplementary material:**

The online version of this article (doi:10.1186/1939-8433-6-33) contains supplementary material, which is available to authorized users.

## Background

Asian cultivated rice (*Oryza sativa* L.) originated from common wild rice (*Oryza rufipogon* Griff.), and their morphological, biochemical and genetic relationships have been analyzed in many studies (Sun et al., 2001; Cai & Morishima 2002). Much of its genetic architecture and phenotypic construction changed during domestication from wild rice. In general, *Oryza sativa* is different from *O. rufipogon* in terms of a number of traits such as plant height, number of spikelets per panicle (SPP), 1000-grain weight, grain shape, and awn. Among these agronomic traits, the SPP and 1000-grain weight are determinants of grain yield (YD).

The number of primary and secondary branches (SBs) strongly influences the average number of SPP (Yamagishi et al., 2002). QTLs for the SPP have been detected using various segregating populations (Kobayashi et al., 2004). Several QTLs for the SPP have also been identified in wild relatives (Thomson et al., 2003; Suh et al., 2005; Onishi et al., 2007). These QTLs are located across the chromosomes and provide valuable information on the genes that control the SPP in different populations. In addition, SPP QTLs have been mapped as a single Mendelian factor (Zhang et al., 2006,2009) and were rarely found on chromosomes 5 and 10 (Thomson et al., 2003; Tan et al., 2008). And these studies showed that the wild rice allele leads to increased or decreased number of SPP.

Increase of the grain weight is a method for increasing rice yield. Genes that affect the grain size have been identified in inter-specific crosses (Xiao et al., 1998; Thomson et al., 2003; Li et al., 2004; Aluko et al., 2004; Brondani et al., 2002). In most cases, wild-type alleles were associated with small grain, whereas cultivar alleles were associated with large grains. Usually, grain size is determined by grain length (GL), width, and thickness. These 3 traits are quantitatively inherited under the control of several or many genes. To date, 5 key genes controlling seed size have been isolated in rice: *GS* 3, *GW* 2, *qSW* 5 or *GW* 5, *GIF1* and *GS5*. (Fan et al., 2006; Song et al., 2007; Shomura et al., 2008; Weng et al., 2008; Li et al., 2011). *GS* 3 has a major effect on seed length, whereas *qSW5/GW5* and *GW2* confer both the seed or grain width (GW) and weight in rice. *GIF1* encodes a cell-wall invertase that is required for carbon partitioning during early grain filling, and the over-expression of *GIF1* by using its native promoter leads to large grains (Wang et al., 2008). Shomura et al. (2008) found that a deletion in *qSW* 5 was associated with grain size owing to an increase in the cell number in the outer glume of the rice spikelet.

A number of QTL studies showed that one genomic region was associated with several traits, especially yield component traits, indicating linkage and/or pleiotropic effects (Xiao et al., 1996; Tian et al., 2006; Tan et al., 2008; Liu et al., 2010). The question of pleiotropy versus tight linkage in these studies should be solved using a large-size population combined with high-density mapping, because its implication is important for improving rice quality and yield. For example, if each of the 2 parents has a TGW-increasing or SPP-increasing QTL that is tightly linked, complementary combination of the 2 beneficial QTLs by using molecular markers could produce higher yields compared to the 2 parents. However, a pleiotropic QTL with opposite effects on the SPP and 1,000-grain weight (TGW) is complicated and challenging in terms of its application to rice improvement.

We conducted this study to characterize the QTL, *qSPP5* in terms of the SPP and to determine its linkage relationship with the grain weight gene, *qTGW5* by using near-isogenic lines that were derived from a cross between Hwayeongbyeo (*O. sativa*) and W1944 (*O. rufipogon*).

## Methods

### Population development

**Figure 1:**
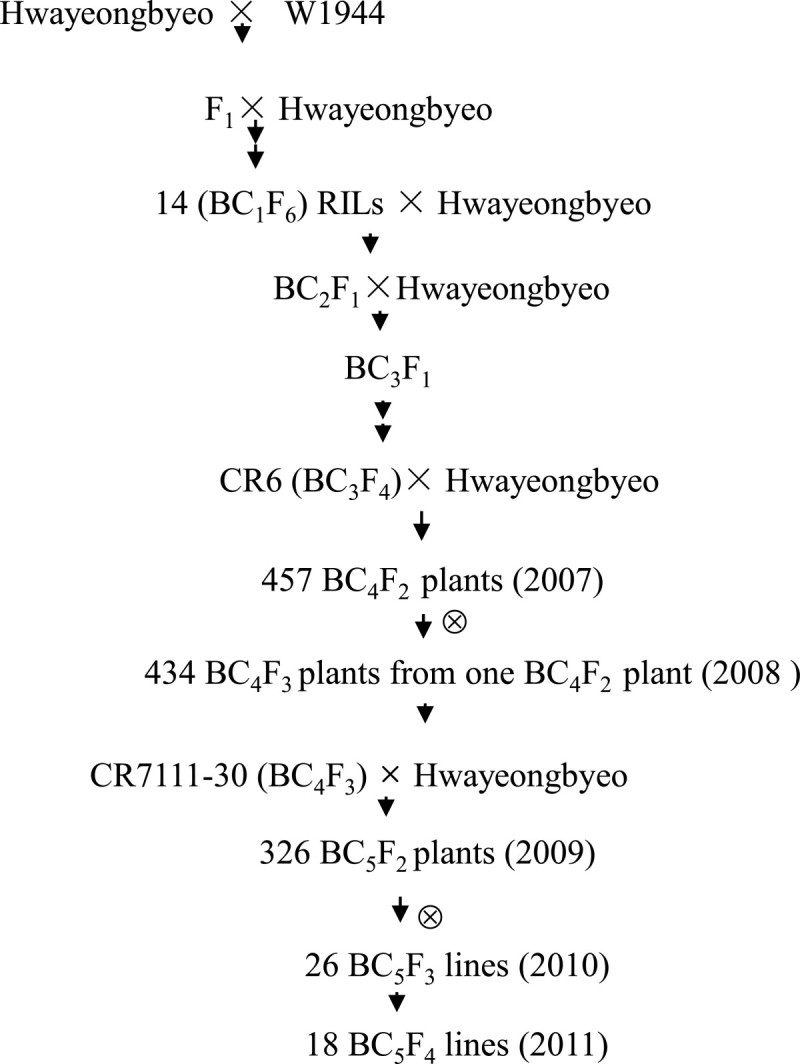
**Development of genetic materials that were used in this study.**

**Figure 2:**
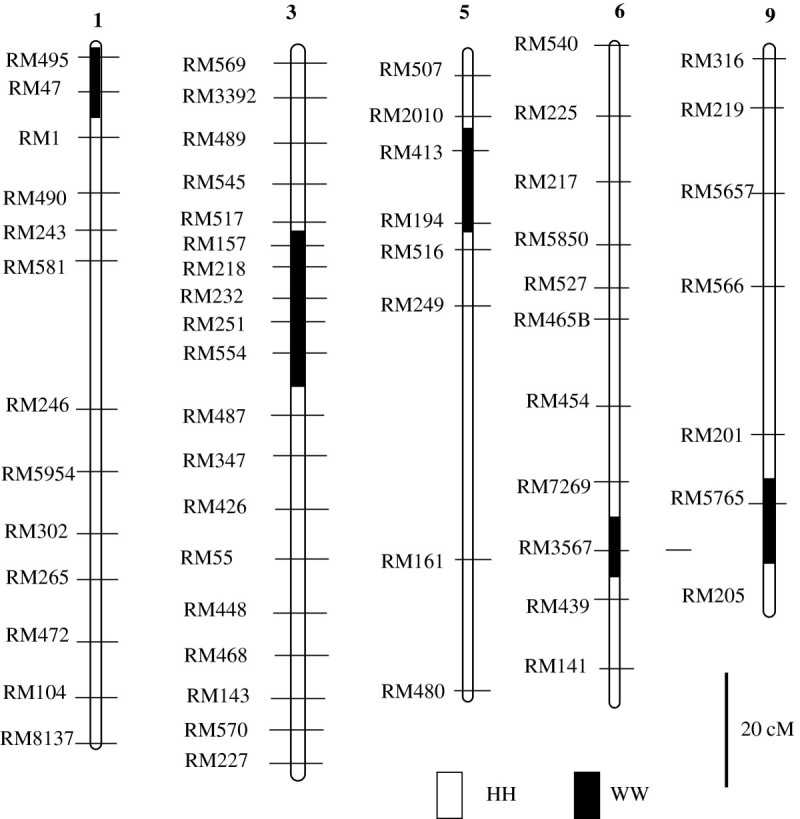
**Graphical genotype of the BC**_**3**_**F**_**4**_**line, CR6.** CR6 had, in total, 5 introgressed segments including the target segment on chromosome 5. HH: Hwayeongbyeo homozygote; WW: W1944 homozygote.

In previous studies, the QTLs for the SPP and GW were detected near the SSR markers RM413 and RM194 on chromosome 5 (Lee et al., 2005; Yuan et al., 2009). The scheme that we used to develop the genetic material is shown in Figure 1. To analyze these QTLs, we selected the BC_3_F_4_ introgression line CR6 as the basis for fine-mapping for the following reasons: (a) it had an *O. rufipogon* introgression across the target region as identified by markers RM413 and RM194 on chromosome 5; (b) it was associated with increased SPP and decreased grain weight; and (c) it had only 4 non-target *O. rufipogon* segments (Figure 2). CR6 was backcrossed to Hwayeongbyeo and then allowed to self to generate a near isogenic line (NIL)-derived BC_4_F_2_ population (457 plants), which showed segregation in the target region on chromosome 5. A single BC_4_F_2_ plant was selected from this population using the same criteria as mentioned above, and the plant was heterozygous across the target region with respect to markers RM413 and RM194 on chromosome 5. The plant was selfed to produce 434 BC_4_F_3_ plants. The QTLs for the SPP and TGW were validated in both of the populations. To further fine map *qTGW* 5, one BC_4_F_3_ plant, CR7111-30, which carried the W1944 homozygous segment for the target region at *qTGW5* locus, was crossed with Hwayeongbyeo to produce a BC_5_F_2_ population with 326 plants. CR7111-30 had no *O. rufipogon* introgression at the non-target regions. Among 326 plants, 127 BC_5_F_2_ plants were evaluated and used for QTL analysis. 26 BC_5_F_2_ plants with informative recombination breakpoints between RM18003 and RM249 were selfed to produce 26 BC_5_F_3_ lines for substitution mapping. Finally, 18 BC_5_F_3_ lines were selected and selfed to produce BC_5_F_4_ lines.

### Phenotypic evaluation

Two populations (BC_4_F_3_ and BC_5_F_2_), 26 BC_5_F_3_ lines, 18 BC_5_F_4_ lines, and the parent Hwayeongbyeo, were grown in the field during the summers of 2008–2011 at the Chungnam National University (36°22′ N, 127°22′ E), Daejeon, Korea. Each plant in BC_4_F_3_ and BC_5_F_2_ was planted 15 cm from the next plant and was spaced at 30 cm between rows. Each line with 25 plants in BC_5_F_3_ and BC_5_F_4_ was represented by a single row of 30-day-old seedlings that were planted 15 cm from the next plant and spaced at 30 cm between rows. The BC_5_F_4_ lines were planted in a completely randomized block design with 3 replications.

### Agronomic traits

The culm length (CL), panicle length, primary branch (PB), secondary branch (SB), SPP, TGW, grain length (GL), grain width (GW), grain thickness (GT), and yield per plant (YD) were evaluated for each plant and line as follows. Five plants from the middle of each line were selected to evaluate the CL and panicle length, and the 2 biggest panicles of 5 plants were selected to evaluate the PB, SB, and SPP. Grains that had hulls were allowed to dry naturally after harvesting, and partial or un-filled seeds were removed by soaking the grains in water. Fully filled seeds were re-dried in an oven at 30°C for 24 h. The TGW was evaluated by measuring the weight of 100 randomly selected, fully filled grains: this method was performed in triplicate and the values were averaged to yield a single mean. The GL, GW, and GT of 100 grains that were fully filled were measured in triplicate using a 150-mm vernier caliper (Mitutoyo Corp., Japan). The YD, which was measured in grams of seed per plant, was determined for 15 plants that were harvested from the middle of 1 plot per block. The TGW and yield per plant were corrected for 12% grain moisture content.

### DNA extraction and simple sequence repeat analysis

DNA was extracted from the fresh leaves of BC_4_F_3_ plants, BC_5_F_2_ plants, and BC_5_F_4_ lines by using the CTAB method described by Causse et al. (1994). SSR primers were synthesized according to an available public rice genomic sequence (http://www.gramene.org/markers/). One primer, INDEL3, in the target region, was designed using primer 3.0 (forward: 5′CATCACTTTCTCTCCTTCCGTTA3′, reverse: 5′TACAGTGTACAGAAAGCTGGTTG3′). A total volume of 20 μL of reaction mixture was composed of 5.0 μL (5 ng/μL) of template DNA, 0.1 μL of Taq polymerase (5 Unit/μL), 0.8 μL of dNTP (2.5 mM each), 1 μL of forward + reverse primer (10 pmol each), 2.0 μL of 10× PCR buffer (10 mM Tris–HCl PH 8.3, 50 mM KCl, 1.5 mM MgCl_2,_ and 0.1% Gelatin), and 11.1 μL of triply distilled water. Amplification was achieved using a Thermo Cycler (Bio-Rad) according to the step-cycle program of denaturation at 94°C for 5 min and then subsequent denaturation performed at 94°C for 1 min, annealing at 55°C for 1 min, and extension at 72°C for 1 min. Steps 2 through 4 were repeated for 35 cycles, in all, followed by a final extension step at 72°C for 5 min. The PCR products were run on a 4% polyacrylamide denaturing gel for 1–2 h at 1800–2000 V, and marker bands were revealed by silver staining (Panaud et al., 1996). 11 SSR markers failed to detect polymorphism in the region between INDEL3 and RM18058 due to genetic similarity between the parents (Lee et al., 2005), and additional genotyping of BC_5_F_4_ lines was conducted with targeted SNP markers. The polymorphism was assayed by direct sequencing of 441-bp (5,697,197 - 5,697,637^th^ position) and 1,162-bp (5,892,883 -5,894,044^th^ position) PCR product generated by two primer pairs in Solgent Co., Korea (http://www.solgent.com). The first (F 5′-gattgacttatatttggacctcc-3′ and R 5′- gtaaacggtagtgttgactgca-3′) and second (F 5′- caaaatgaatcggccgaagcac -3′ and R 5′- cagaccagtgtgaagaggagg -3′) primers were designed according to the sequences of the *O. rufipogon* and Nipponbare (http://rgp.dna.affrc.go.jp/E/IRGSP/Build5/build5.html). The sequence information of *O. rufipogon* in the target region was provided by Dr. S.R. McCouch at Cornell University. The first SNP, hereafter referred to as SNP-1 which occurs at the 5,697,388^th^ position based on the Nipponbare sequence (http://rgp.dna.affrc.go.jp/E/IRGSP/Build5/build5.html) is characterized by nucleotide T in Hwayeongbyeo but nucleotide C in *O. rufipogon*. The second SNP, SNP-2 which occurs at the 5,893,072^th^ position based on the Nipponbare sequence (http://www.gramene.org) is characterized by nucleotide thymine (T) in Hwayeongbyeo but nucleotide cytosine (C) in *O. rufipogon*.

### Statistical analysis

One-way ANOVA was performed to determine the effect of each marker on each of the traits. Phenotypic means of 3 genotypes- Hwayeongbyeo and W1944 homozygotes and heterozygotes- were compared using Student’s *t*-test, and a probability level of 0.5% was used as the threshold for detecting a QTL. The proportion of total phenotypic variance that was explained by each QTL was calculated as an R^2^ value by carrying out regression analysis using each marker/phenotype combination. QTLs were fine-mapped by comparing the phenotypic means of 3 genotypes of recombinants within the target region by using the SAS statistical software package (SAS Institute, Cary, NC, USA).

## Results

### Characteristics of CR6

Two parents, CR6 and Hwayeongbyeo, showed significant differences in 6 traits (Table 1). Hwayeongbyeo exhibited less number of SPP but higher TGW than CR6 did. The GW of Hwayeongbyeo was larger than that of CR6, whereas no significant differences in the GT and GL were detected between the 2 parents (data not shown). Moreover, no significant difference was observed for days to heading and spikelet fertility (data not shown).

**Table 1:** **Comparison of 6 agronomic traits between Hwayeongbyeo and CR6**

Trait^#^	Hwayeongbyeo	CR6	Difference^@^
SPP	118.2 ± 12.3	142.4 ± 15.1	**
TGW	25.5 ± 1.8	23.1 ± 1.3	**
GW	1.58 ± 0.19	1.45 ± 0.18	**
SB	20.4 ± 5.2	27.2 ± 5.9	**
PL	20.3 ± 2.6	22.1 ± 2.9	*
CL	83 ± 5.2	87 ± 4.2	**

^#^SPP, TGW, GW, SB, PL, and CL: spikelets per panicle, 1,000-grain weight, grain width, secondary branches per panicle, panicle length, and culm length, respectively.

^@^ *, **: Significant at *P* = 0.05 and 0.01, respectively.

### Frequency distribution of the BC_5_F_2_ population

**Figure 3:**
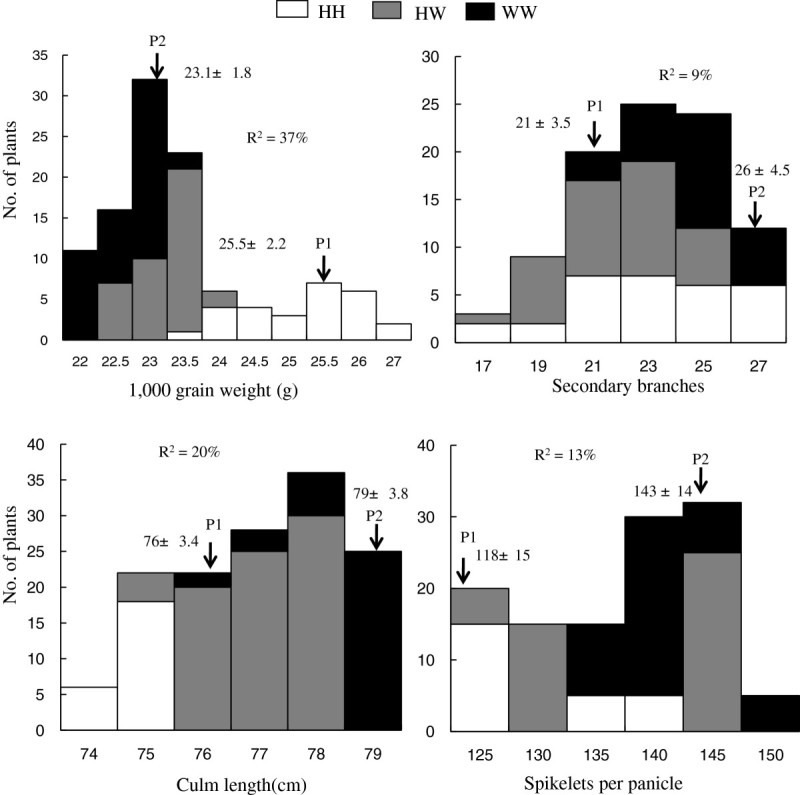
**Frequency distribution of 4 traits in the BC**_**5**_**F**_**2**_**population.** Three genotypes of the Hwayeongbyeo homozygous and heterozygous and *O. rufipogon* homozygous classes were identified using the simple sequence repeat marker RM194. HH: Hwayeongbyeo homozygote; WW: W1944 homozygote; and HW: heterozygote. P_1_ and P_2_ denote Hwayeongbyeo and CR7111-30, respectively.

Frequency distributions of phenotypes for the TGW, SPP, SB, and CL of the BC_5_F_2_ population are shown in Figure 3. The TGW showed a bimodal distribution with 23.5 as the trait value boundary. The other 3 traits exhibited continuous and normal distributions. The distribution indicated that the *O. rufipogon* segment was associated with increases in the SB and SPP, and decreases in the TGW in the Hwayeongbyeo background. The genotypes of the BC_5_F_2_ plants were determined at RM194 and the phenotypic variances that were explained by the marker were 37.0%, 13.9%, 9%, and 20.0%, respectively.

### QTLs in BC_4_F_3_, BC_5_F_2_, BC_5_F_3_, and BC_5_F_4_

The possibility of the effect of non-target regions on SPP and other traits can be excluded because CR7111-30 had no *O. rufipogon* introgression at the non-target regions. Two SSR markers, RM413 and RM194, were used to genotype the BC_4_F_3_ and BC_5_F_2_ generations. The QTLs for the TGW, SPP, CL, PL, SB, and GW were all linked to RM194 (Table 2). Seven markers were used to genotype the BC_5_F_3_ and BC_5_F_4_ populations. The orientations and distances between the markers were based on Nipponbare sequence information (http://www.gramene.org/markers/microsat/). QTL analysis for 5 traits revealed that there was a significant peak near the marker RM194 for the TGW, CL, SPP, SB, and GW, and a peak near RM18076 (Table 2). The phenotypic variance that was explained by each QTL was 9.4-79.0%. This result indicates that this region was a QTL cluster. The SPP for the Hwayeongbyeo homozygous class (HH), the heterozygous class (HW), and the *O. rufipogon* class (WW) were 119, 147, and 141 at RM18076 in BC_5_F_4_, respectively. TGW for the Hwayeongbyeo HH, HW, and the *O. rufipogon* class WW were 27.1, 25.4, and 24.2 respectively.

**Table 2:** **QTLs detected in the BC**_**4**_ **F**_**3,**_ **BC**_**5**_ **F**_**2,**_ **BC**_**5**_ **F**_**3,**_ **and BC**_**5**_ **F**_**4**_ **generations**

Trait^$^	QTL	Marker	Pop.	P	R^2^	Phenotypic mean ± s.d.^%^		
						HH	HW	WW
TGW	qTGW5	RM194	BC_4_F_3_	0.0001	45.9	26 ± 1.3(108)^#^	24 ± 1.7(210)	23 ± 1.2(113)
		RM194	BC_5_F_2_	0.0001	37.1	26 ± 0.8(28)	24 ± 1.1(55)	23 ± 0.8(44)
		RM194	BC_5_F_3_	0.0001	64.8	25 ± 0.4(5)	24 ± 0.6(11)	23 ± 0.4(10)
		RM194	BC_5_F_4_	0.0001	79.0	25 ± 0.7(6)	24 ± 0.9(6)	23 ± 0.6(6)
SPP	qSPP5	RM194	BC_4_F_3_	0.01	9.7	123 ± 22	145 ± 25	144 ± 21
		RM194	BC_5_F_2_	0.01	13.0	126 ± 17	140 ± 17	140 ± 16
		RM194	BC_5_F_3_	0.01	19.5	133 ± 11	148 ± 12	146 ± 12
		RM18058	BC_5_F_4_	0.005	33.0	125 ± 10	148 ± 12	150 ± 12
SB	qSB5	RM194	BC_4_F_3_	0.01	9.0	24 ± 0.8	25 ± 1.2	26 ± 1.1
		RM194	BC_5_F_2_	0.005	9.0	23 ± 0.8	26 ± 1.0	26 ± 0.9
		RM194	BC_5_F_3_	0.005	20.9	23 ± 0.7	24 ± 1.0	25 ± 0.8
		RM194	BC_5_F_4_	0.0001	35.7	23 ± 0.8	26 ± 1.0	27 ± 0.9
CL	qCL5	RM194	BC_4_F_3_	0.01	9.4	77 ± 3.1	78 ± 3.2	79 ± 2.8
		RM194	BC_5_F_2_	0.005	20.0	75 ± 2.6	78 ± 3.0	78 ± 2.9
		RM194	BC_5_F_4_	0.005	21.5	79 ± 1.9	83 ± 2.0	83 ± 1.9
GW	qGW5	RM194	BC_5_F_4_	0.0001	62.0	1.58 ± 0.10	1.49 ± 0.10	1.45 ± 0.11

^$^TGW: 1,000-grain weight; SPP: number of spikelets; SB: number of secondary branches; CL: culm length; and GW: grain width. ^%^HH: Hwayeongbyeo homozygotes; HW: heterozygotes; and WW: *O. rufipogon* homozygotes. ^#^Numbers in parenthesis indicate the number of plants or lines.

A strong positive correlation (*r* = 0.845, *P* < 0.001) was observed between the GW and TGW in BC_5_F_4_, indicating that the variation in the GW was associated with that in the TGW at this locus (data not shown).

### Substitution mapping

**Figure 4:**
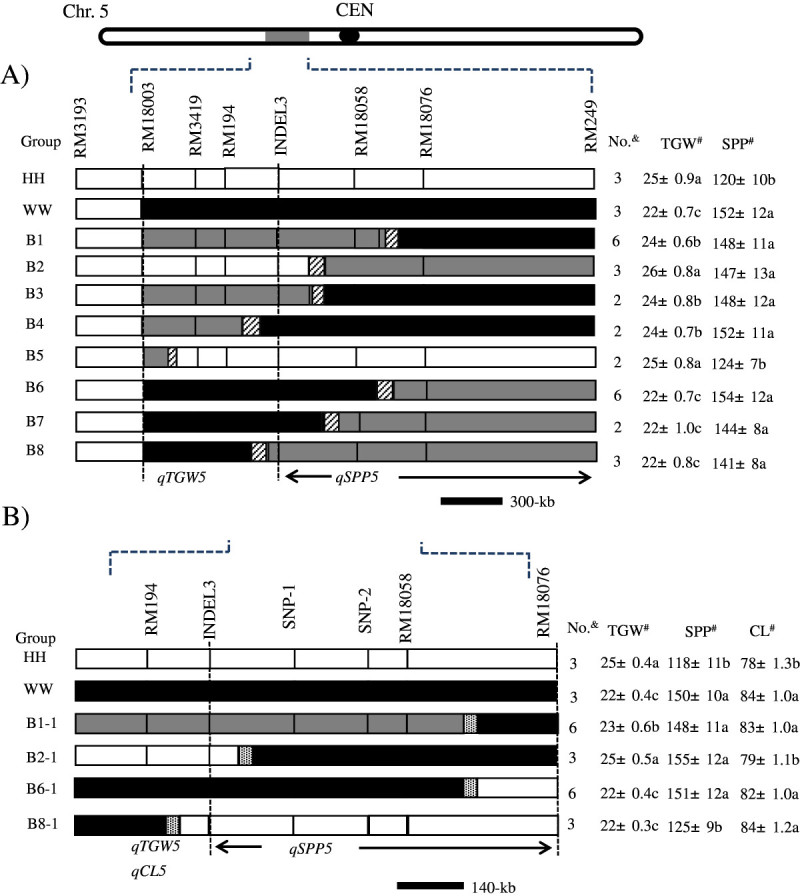
**Substitution mapping of** ***qSPP5*** **and** ***qTGW5*** **using two populations. A)** Graphical genotypes of the BC_5_F_3_ lines that were used for the substitution mapping of *qSPP5* and *qTGW5*. The white portions of the graph indicate homozygous Hwayeongbyeo chromosome segments, the black regions indicate homozygous *O. rufipogon* chromosomes, the gray areas indicate heterozygous regions, and the slashed areas are regions where crossing-over occurred. The table on the right of the graphical genotypes shows the mean values of 2 traits for each genotype. The broken vertical lines define the interval that contained 2 QTLs. ^&^Number of lines in each group. ^#^The numbers that are followed by different letters in each column were significantly different according to Tukey’s HSD test at 5%. **B)** Graphical genotypes of the BC_5_F_4_ lines that were used for the substitution mapping of *qSPP5, qTGW5* and *qCL5*. HH and WW in the Group indicate Hwayeongbyeo and CR7111-30, respectively.

Substitution mapping was carried out for *qTGW5* and *qSPP5* by using the BC_5_F_3_ and BC_5_F_4_ populations (Figure 4). Seven markers were used to screen 26 BC_4_F_3_ lines, and these lines were evaluated for the TGW and SPP. The 26 lines were classified into 8 groups based on the genotypes of the SSR markers. The mean phenotypic values of the TGW and SPP for each group were compared to those of the controls, Hwayeongbyeo and CR7111-30. A comparison of the genotypes of recombinants delimited the *qTGW5* locus between markers INDEL3 and RM18003 based on the finding that the TGW of the B5 lines with a recombination breakpoint between RM18003 and RM3419 did not significantly differ from that of Hwayeongbyeo but was higher than that of CR7111-30. Moreover, the TGW of B8 lines with a recombination breakpoint between INDEL3 and RM194 did not significantly differ from that of CR7111-30 but was lower than that of Hwayeongbyeo.

For the *qSPP5* locus, group B5 had a significantly lower SPP than CR7111-30 did. The SPP of group B2 with a breakpoint between markers RM18058 and INDEL3 did not significantly differ from that of CR7111-30 but was significantly higher than that of Hwayeongbyeo. These results imply that *qSPP5* was located downstream of INDEL3. The parents and the heterozygote class (B1 and B3) showed significant differences in the TGW, indicating that the TGW gene was under additive genetic control. The heterozygote classes (B6 and B7) and CR7111-30 showed significantly higher SPP than Hwayeongbyeo did, indicating that the SPP gene was under dominant genetic control.

To further define the linkage relationship between *qSPP5* and *qTGW5*, we self-crossed 18 BC_5_F_3_ plants that were selected from B1, B2, B6, and B8 to produce 18 BC_5_F_4_ lines and evaluated them in terms of the TGW, SPP and CL (Figure 4B). Also, BC_5_F_4_ lines were genotyped with two SNP markers, SNP-1 and SNP-2. The mean phenotypic values of the SPP and TGW for each group in BC_5_F_4_ were compared to those of the controls, Hwayeongbyeo and CR7111-30. The TGW of B2-1 was significantly higher than that of B1-1 and B6-1, which suggests that the *qTGW5* allele was located in the upstream region of SNP-1. The TGW of B8-1 was significantly lower than that of Hwayeongbyeo, which suggests that the *qTGW5* allele was located in the upstream region of INDEL3. For *qSPP5*, B2-1 significantly differed from Hwayeongbyeo in SPP, which indicated that the *qSPP5* allele was located in the downstream region of INDEL3. The number of SPP of B6-1 was significantly higher than that of Hwayeongbyeo, which suggests that *qSPP5* was located in the upstream region of RM18076. The group B8-1 did not show difference in the number of SPP compared to Hwayeongbyeo, and this indicated that the QTLs for the SPP and TGW were different. We found that *qTGW5* was located in the upstream region of INDEL3, whereas *qSPP5* was located in about 860-kb interval between INDEL3 and RM18076 based on the Nipponbare sequence (http://www.gramene.org). To map the *qCL5*, the same procedure was applied and *qCL5* was located in the upstream of INDEL3.

### *O. rufipogon* contains Kasalath-type qSW5

The *qTGW* 5 seemed to be the same gene as *qSW5* based on its position (Shomura et al., 2008). Three allelic types at the *qSW5* locus exist: Kasalath-type, *Indica* II-type, and Nipponbare-type. Of these, the Kasalath-type allele is functional and the Nipponbare-type is a loss-of-function allele. A 1212-bp deletion at the *qSW5* locus in Nipponbare was associated with an increase in the GW, as compared to Kasalath. One hundred eighty rice cultivars were genotyped at the *qSW5* locus by using the primers, and they were divided into 3 types: Kasalath-type, *Indica II*-type, and Nipponbare-type (Song et al., 2011). To determine the allele type of *O. rufipogon* at *qGW5*, we genotyped *O. rufipogon* by using the N1212del. The results showed that Hwayeongbyeo and W1944 had the Nipponbare-type and Kasalath-type alleles, respectively (data not shown). This result seemed to confirm that *qTGW5* in this study was the same gene as *qSW5*.

### Impact of the QTL cluster on the YD per plant

Two BC_5_F_4_ NILs, B8-1 (*O. rufipogon* homozygous at *qTGW5* and Hwayeongbyeo homozygous at *qSPP5*) and B2-1 (Hwayeongbyeo homozygous at *qTGW5* and *O. rufipogon* homozygous at *qSPP5*), were used for yield trials together with the parental controls in 2011. The trials were conducted using a completely randomized block design with 3 repetitions. The results show that the average YD per plant of B2-1 was 15.3% higher than that of B8-1 (*P* ≤ 0.02). The average YD per plant of B2-1 was 7.3% higher than that of Hwayeongbyeo (*P* = 0.06), although the difference was not significant at *P =* 0.05 (Table 3).

**Table 3:** **Comparison of grain yield per plant between 2 QTL-NILs and their parents**

Line	Trait mean ± s.d.^@^		
	DTH	CL	YD
Hwayeongbyeo	98a^+^, a^#^	83b, b	26.0 ± 1.3 ab, b
CR7111-30	98a, a	86a, a	25.7 ± 1.4 bc, b
B2-1	98a, a	82b, b	27.9 ± 1.6 a, a
B8-1	97a, a	86a, a	24.0 ± 1.5 d, c

^@^DTH: days to heading; CL: culm length; and YD: yield per plant.

^*+, #*^The numbers that are followed by the same letters were not significantly different according to Tukey’s HSD test at 5% (^+^) and 10% (^#^), respectively.

## Discussion

The original target of this study was the QTL for the TGW, which was *qTGW5* mapped on chromosome 5 (,^2005^). During the process of fine-mapping this trait, the QTLs for the SPP, SB, and CL were consistently detected in the same region. The QTL for the SPP was detected near the SSR markers RM413 and RM194 on chromosome 5, and the coefficient of determination was low being 3.7% (Lee et al., 2005). However, the effect of *qSPP5* was not strong to be detected by both interval mapping and single-point analysis near the same SSR markers (Yuan et al., 2009). It is likely that *qSPP5* is a minor QTL and not stable. Substitution lines confirmed that the QTL for TGW resided in the 165-kb region and that the additional 4 QTLs were co-localized near *qTGW5*.

A number of QTLs for the SPP have been identified using inter- (Thomson et al., 2003; Tian et al., 2006) and intra-specific populations (Cui et al., 2003; Lu et al., 1997), and these QTLs were located on all of the rice chromosomes. However, a few studies reported on a QTL that is associated with the SPP and is located on chromosome 5 by using inter-specific populations (Lee et al., 2005; Tian et al., 2006; Tan et al., 2008). Based on the finding that the wild alleles increased the number of SPP and decreased the TGW, and their map position, it appears that *qGPA* 5 reported by Tian et al. (2006) and *spp5.1* detected by Tan et al. (2008) are allelic to *qSPP5* in this study. It is interesting that the QTL for the SPP was detected exclusively using introgression lines from crosses between cultivars and Asian common wild rice (Lee et al., 2005; Tian et al., 2006; Tan et al., 2008). One possible reason is that the effect of these QTLs was not so strong that they could not be detected in primary mapping populations such as F_2_ and RILs (Xiao et al., 1996) because *qGPA* 5 (Tian et al., 2006), *spp5.1* (Tan et al., 2008), and *qSPP5* in this study were detected in the introgression lines population. Because the SPP is inherited quantitatively, this trait is tractable to genetic analysis via the development of high-resolution NILs. NILs that block genetic background noise would be useful for validating minor QTLs and mapping them as a single Mendelian factor (Xie et al., 2008). As documented in this study, the R^2^ values steadily increased with progressive generations of backcrossing from 9.7% for the BC_4_F_3_ generation to 33.0% for the BC_5_F_4_ generation of NILs. As the number of spurious donor (i.e., *O. rufipogon*) introgressions in the genetic background decreased and the linkages between the markers and the target gene(s) increased, the proportion of phenotypic variation that could be explained by the markers greatly enhanced.

Whether similar genomic locations of QTLs that affect different traits are attributable to the pleiotropy of a single gene or the tight linkage of several genes that individually influence specific traits has been a topic of debate. In a previous study by Xiao et al. (1996), pleiotropy was suggested for 3 chromosomal regions that were simultaneously associated with the TGW and grains per plant or the TGW and grains per panicle. These yield components showed highly negative correlations, and 3 significant QTLs that were associated with the TGW were mapped to the same positions as 3 QTLs that affect grains per plant and grains per panicle. In this study, one genomic region was associated with more than one trait, which indicated the existence linkage and/or pleiotropic effects. Liu et al. (2010) mapped the QTLs for grain weight *TGW3b* and the SPP *SPP3b* to a 2.6-cM interval between RM15885 and W3D16. At this QTL region, the Teqing allele was associated with an increase in the SPP and a decrease in the TGW, and no conclusion could be drawn about whether one pleiotropic QTL or two linked QTLs were located within the interval. Bai et al. (2011) also reported that 2 QTLs, *qssp8* and *tgw8*, which are located between RM502 and RM264, might be the same gene. In our study, we demonstrated that 2 tightly linked QTLs, *qSPP5* and *qTGW5*, control the SPP and grain weight, respectively. In this regard, the question of pleiotropy versus tight linkage in these studies remains to be resolved using larger populations and high-density mapping.

A high YD is one of the most important goals of rice breeding programs. Much attention has been focused on the genetic bases of the SPP and TGW because of their importance in determining rice yield. In this study, the effect of the detected QTL *qSPP5* was confirmed by the increase in the SPP of the NILs. *qSPP5* is a minor QTL that exhibits a small additive effect of approximately 10–15 spikelets. The high number of SPP in the NIL was mainly attributed to the increased number of SBs. The finding that yield per Hwayeongbyeo plant could be improved by introgressing *qSPP5*, which is a QTL for the SPP from *O. rufipogon*, demonstrates the existence of a complementary combination between 2 linked QTLs, *qTGW5* and *qSPP5*, with the aid of molecular markers. Specifically, the pyramiding of the Hwayeongbyeo allele at *qTGW5* and the *O. rufipogon* allele at *qSPP5* should produce a higher yield compared to the parental genotypes. As expected, the NIL with the wild allele at *qTGW5* and the Hwayeongbyeo allele at *qSPP5* had lower yields compared to Hwayeongbyeo. The data presented in this study clearly indicate the linkage of *qSPP5* and *qTGW5* although additional experiments using lines from a cross between two separate lines each segregating at one QTL region but fixed at another QTL might be necessary to further confirm their linkage. Based on the finding that the *O. rufipogon* alleles for the SPP are beneficial in the *japonica* and *indica* cultivar backgrounds (Lee et al., 2005; Tian et al., 2006; Tan et al., 2008), the *qSPP5* allele could be valuable gene (s) for improving rice yields.

QTL mapping indicated the existence of five QTLs in this region across different generations and substitution mapping confirmed the linkage of QTLs for SPP and TGW. The finding that the gene (s) affecting two traits, SPP and SP were mapped to the same region and the same direction of the genetic effect with *O. rufipogon* alleles increasing trait values across different generations implies that this locus was associated with panicle structure with pleiotropic effects. Similar results were reported in the study by Ohsumi et al. (2011) that Habataki alleles of *qSBN1* and *qPBN6* increased spikelet number on secondary rachis branches and primary rachis branches in the Sasanishiki genetic background. A strong positive correlation (*r* = 0.845, *P* < 0.001) between the GW and TGW in BC_5_F_4_ seems to suggest that the variation in the GW was associated with that in the TGW at this locus which controls grain morphology traits (data not shown). This result is also consistent with the report by Weng et al. (2008) that *GW5* is associated with rice grain width and weight.

Several QTLs that control the SPP have been cloned using NILs (Xue et al. 2008; Miura et al. 2010). In the present study, *qSPP5* was responsible for 33.0% of the phenotypic variance. No QTL around the *qSPP5* region has been cloned to date. It would be interesting to clone *qSPP5* to examine the functional relationships of the genes that control the SPP and to determine how they interact with other genes/alleles in various genetic backgrounds. The BC_5_F_4_ NILs that were developed in this study could be good materials for further fine mapping and cloning of *qSPP5*.

## Conclusion

In this study, we demonstrated that 2 QTLs, *qSPP5* for spikelets per panicle (SPP) and *qTGW5* for grain weight (TGW), are tightly linked on chromosome 5. Based on the finding that the *O. rufipogon* allele for the SPP was beneficial in the *japonica* and *indica* cultivar backgrounds, the *qSPP5* allele could be valuable for improving rice yields. In addition, the NIL populations and molecular markers are useful for cloning *qSPP5*.

## Authors’ original submitted files for images

Below are the links to the authors’ original submitted files for images. Authors’ original file for figure 1 Authors’ original file for figure 2 Authors’ original file for figure 3 Authors’ original file for figure 4
