# Supplementary material for: QTL mapping reveals a tight linkage between QTLs for grain weight and panicle spikelet number in rice
Source: Rice (N Y). 2013 Nov 28;6:33. doi: 10.1186/1939-8433-6-33 (PMC4883721; doi:10.1186/1939-8433-6-33)
Supplement: Supplementary file 1 — Authors’ original file for figure 1 [file 12284_2013_66_MOESM1_ESM.pdf]

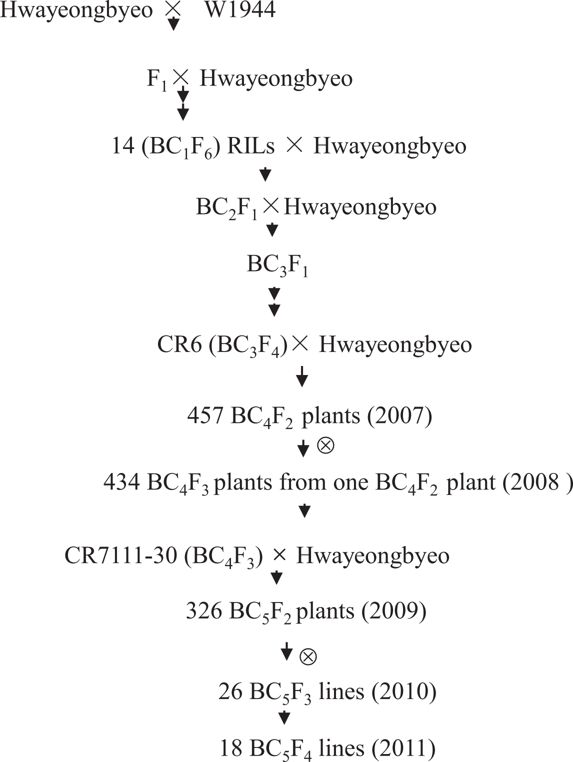
Hwayeongbyeo × W1944
F_1 × Hwayeongbyeo
14 (BC_1F_6) RILs × Hwayeongbyeo
BC_2F_1 × Hwayeongbyeo
BC_3F_1
CR6 (BC_3F_4) × Hwayeongbyeo
457 BC_4F_2 plants (2007)
⊗
434 BC_4F_3 plants from one BC_4F_2 plant (2008)
CR7111-30 (BC_4F_3) × Hwayeongbyeo
326 BC_5F_2 plants (2009)
⊗
26 BC_5F_3 lines (2010)
18 BC_5F_4 lines (2011)
