# Supplementary figures and images for: QTL mapping reveals a tight linkage between QTLs for grain weight and panicle spikelet number in rice
Source: Rice (N Y). 2013 Nov 28;6:33. doi: 10.1186/1939-8433-6-33 (PMC4883721; doi:10.1186/1939-8433-6-33)

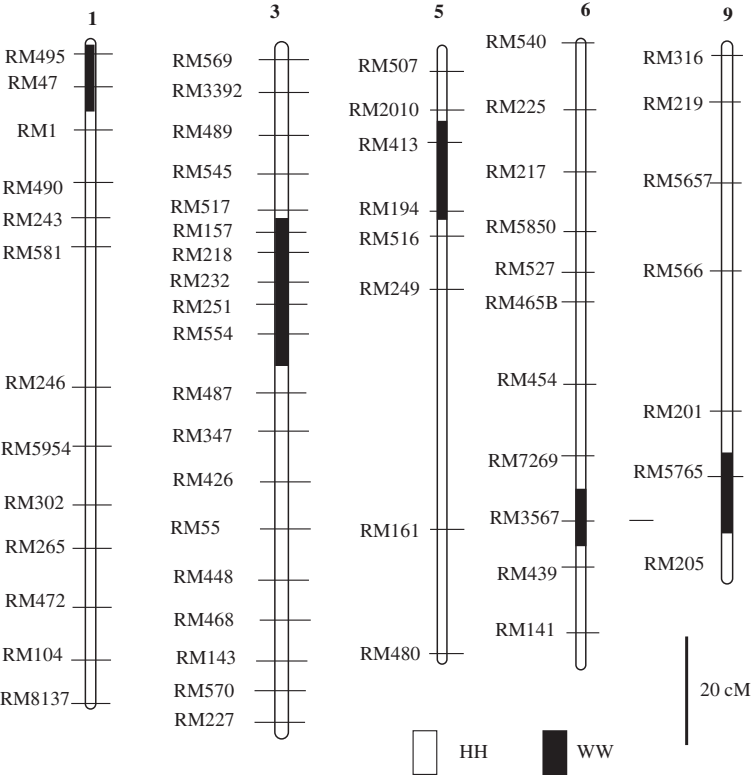

Supplement: Supplementary file 2 — Authors’ original file for figure 2 [file 12284_2013_66_MOESM2_ESM.pdf]

HH HW WW

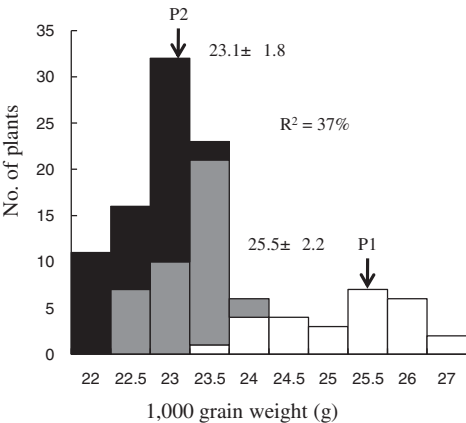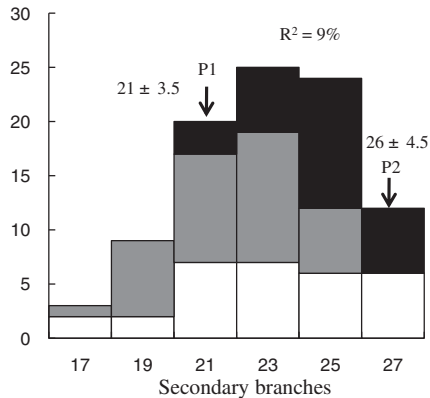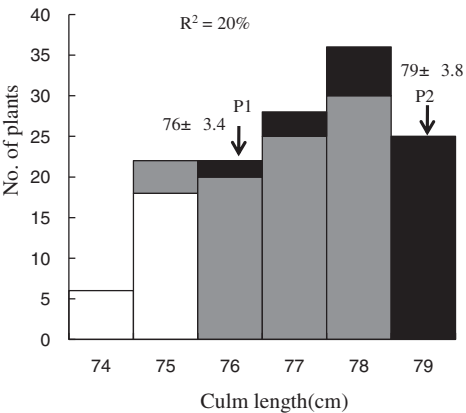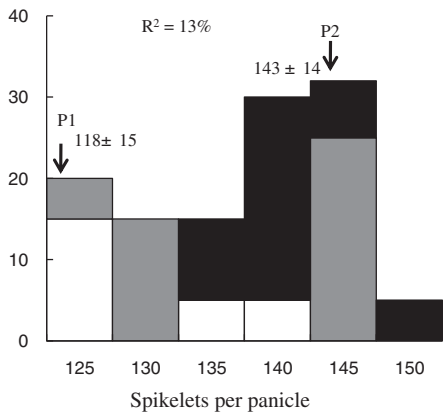

Supplement: Supplementary file 3 — Authors’ original file for figure 3 [file 12284_2013_66_MOESM3_ESM.pdf]

A)

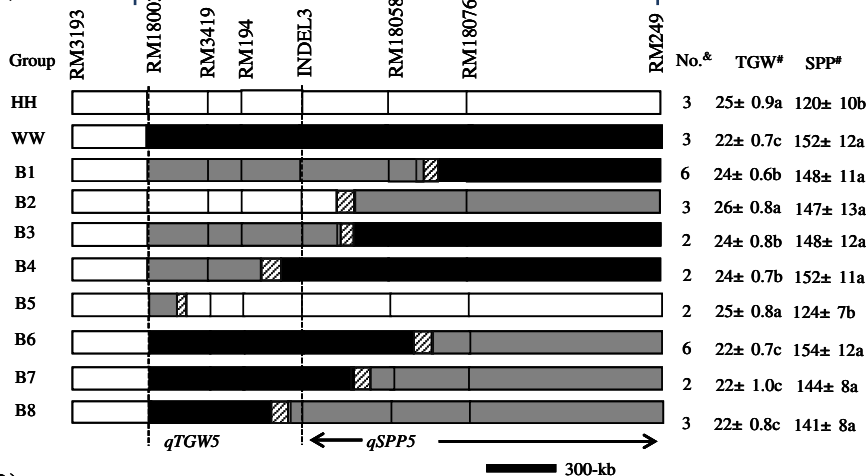

B)

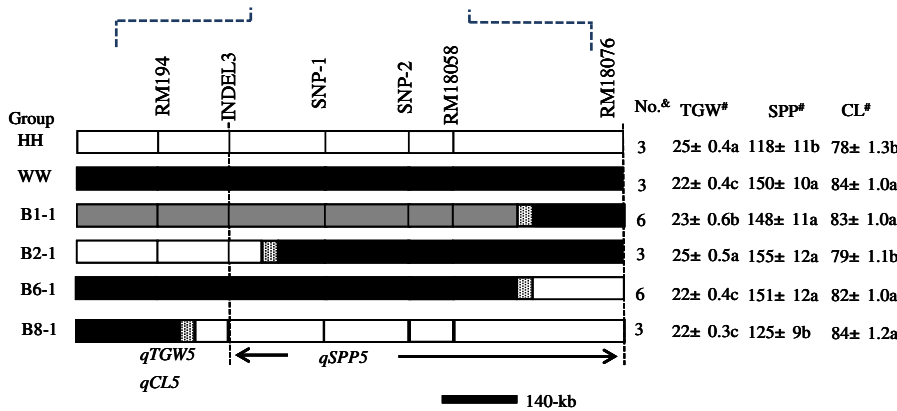

Supplement: Supplementary file 4 — Authors’ original file for figure 4 [file 12284_2013_66_MOESM4_ESM.pdf]
